# The New Maudsley Model for carers of adults with Anorexia Nervosa treated with Enhanced Cognitive Behaviour Therapy: the role of parental support

**DOI:** 10.1192/j.eurpsy.2026.12033

**Published:** 2026-07-31

**Authors:** C. Dani, E. Rossi, E. Cassioli, V. Z. Cordasco, A. Roscioli, G. Selvi, L. Tarchi, L. Zompa, S. Moretti, M. R. Troiani, S. Lucarelli, V. Cardi, V. Ricca, G. Castellini

**Affiliations:** 1 Department of Health Sciences, University of Florence; 2 UFS Eating Disorders ASL Toscana Centro, Florence; 3 Department of General Psychology, University of Padova, Padova, Italy; 4 Centre for Research in Eating and Weight Disorders, Institute of Psychiatry, Psychology and Neuroscience, King’s College, London, United Kingdom

## Abstract

**Introduction:**

The impact of the add-on of New Maudsley Model (NMM) training workshops for carers of individuals with eating disorders (EDs) on clinical outcomes in adults with anorexia nervosa (AN) undergoing Enhanced Cognitive Behaviour Therapy (CBT-E) remains unexplored.

**Objectives:**

This study aimed to compare the outcome of individuals treated with CBT-E whose parents attended the NMM workshops with those whose parents did not receive parental support.

**Methods:**

A longitudinal study was conducted on 186 adult female individuals with AN, divided into two groups based on parental participation in NMM workshops. Psychopathology was assessed up to one-year follow-up. Baseline differences were analyzed using Analysis of Covariance (ANCOVA), while longitudinal changes were evaluated with Generalized Additive Mixed Models (GAMM).

**Results:**

At admission, individuals in the NMM group had higher levels of ED psychopathology, body uneasiness, and alexithymia. At one-year follow-up, they showed a greater increase in body mass index and more significant reductions in ED psychopathology, general psychopathology, body uneasiness, emotional dysregulation, and alexithymia.

**Conclusions:**

Parental participation in NMM workshops was associated with more favorable treatment outcomes in adults with AN undergoing CBT-E. These findings suggest the potential relevance of integrating family participation into adult AN treatment.

**Disclosure of Interest:**

None Declared

